# Osteoporosis: Causes, Mechanisms, Treatment and Prevention: Role of Dietary Compounds

**DOI:** 10.3390/ph17121697

**Published:** 2024-12-16

**Authors:** Kristine Stromsnes, Cristian Martinez Fajardo, Silvana Soto-Rodriguez, Erika Ria Ulrika Kajander, Remus-Iulian Lupu, Monica Pozo-Rodriguez, Balma Boira-Nacher, Maria Font-Alberich, Marcos Gambini-Castell, Gloria Olaso-Gonzalez, Maria-Carmen Gomez-Cabrera, Juan Gambini

**Affiliations:** 1 Department of Physiology, Faculty of Medicine, University of Valencia and CIBERFES, Fundación Investigación Hospital Clínico Universitario/INCLIVA, 46010 Valencia, Spain; kristine.stromsnes@uv.es (K.S.); silvana.soto@uv.es (S.S.-R.); erika.kajander@helsinki.fi (E.R.U.K.); riulian@incliva.es (R.-I.L.); mariafont2001@gmail.com (M.F.-A.); marcosrider12@gmail.com (M.G.-C.); gloria.olaso@uv.es (G.O.-G.); carmen.gomez@uv.es (M.-C.G.-C.); 2 Instituto Botánico, Universidad de Castilla-La Mancha, Campus Universitario s/n, 02071 Albacete, Spain; cristian.martinez@uclm.es; 3 CIC bioGUNE, Bizkaia Technology Park, Building 801A, 48160 Derio, Spain; mpozo@cicbiogune.es; 4 Department of Physical Education and Sports, Faculty of Sports Science, Sport and Health University Research Institute (iMUDS), University of Granada, 18071 Granada, Spain; balmaboira@ugr.es

**Keywords:** osteoporosis, risk factors, polyphenols, disease prevention, lifestyle, bone regeneration, pharmacological treatments, oxidative stress, nutraceuticals, diet

## Abstract

Osteoporosis is a chronic disease that is characterized by a loss of bone density, which mainly affects the microstructure of the bones due to a decrease in bone mass, thereby making them more fragile and susceptible to fractures. Osteoporosis is currently considered one of the pandemics of the 21st century, affecting around 200 million people. Its most serious consequence is an increased risk of bone fractures, thus making osteoporosis a major cause of disability and even premature death in the elderly. In this review, we discuss its causes, the biochemical mechanisms of bone regeneration, risk factors, pharmacological treatments, prevention and the effects of diet, focusing in this case on compounds present in a diet that could have palliative and preventive effects and could be used as concomitant treatments to drugs, which are and should always be the first option. It should be noted as a concluding remark that non-pharmacological treatments such as diet and exercise have, or should have, a relevant role in supporting pharmacology, which is the recommended prescription today, but we cannot ignore that they can have a great relevance in the treatment of this disease.

## 1. Introduction

Osteoporosis is a chronic disease that is characterized by a loss of bone density, which mainly affects the microstructure of the bones due to a decrease in bone mass, thereby making them more fragile and susceptible to fractures. Osteoporosis is currently considered one of the pandemics of the 21st century, affecting around 200 million people. Its most serious consequence is an increased risk of bone fractures, thus making osteoporosis a major cause of disability and even premature death in the elderly [1,2]. Osteoporotic fractures also affect quality of life and are associated with life-threatening complications, i.e., pneumonia, skin infections and sepsis [3]. Although low bone density is a major indicator of osteoporosis, bone density is highly affected by body mass. One useful approach to compare populations is by bone fracture rate [2], which increases exponentially with age, and in women, the prevalence is higher (Table 1) [4].

The incidence of osteoporosis varies geographically, and it has been shown that Scandinavian countries have the highest rate of fractures, with an increase in fractures in favor of latitudes closer to the North Pole, possibly explained by the low serum levels of vitamin D [5]. Geographical variations are thought to include several causes, such as genetic predisposition and environmental factors [1]. Although women are generally more susceptible to osteoporosis, fracture rates also differ among ethnicities. According to the results of Salari N et al., the prevalence of osteoporosis in Africa was found to be higher than in other continents. However, it should be noted that the number of epidemiological studies on osteoporosis in Africa is limited. The prevalence of osteoporosis is much lower in America than in Europe and Asia. Caucasians and Asians often have lower bone density [1], and hip fracture rates are the highest in Northern European countries [6]. This might be explained by different lifestyles, nutrition and anatomy, as well as by genetic predisposition [1,5]. Nevertheless, the prevalence of osteoporosis is expected to rise with the aging population and lifestyle changes [1,2]. To diagnose osteoporosis, body density is measured by performing a DEXA scan. The bone density of the individual is then compared to that of a healthy young adult by calculating the standard deviation, thereby generating what is called a T-score. A T-score ≥ −1.0 indicates normal bone density, a T-score < −1.0 and >−2.5 indicates low bone mass and is defined as osteopenia and finally a T-score ≤ −2.5 shows bone loss and is defined as osteoporosis [7,8].

There are several models to investigate osteoporosis and its consequences, and those present in this review include the following: ovariectomized rats and mice [9,10], mesenchymal cells derived from the bone marrow of mice [11], such as osteoblasts, osteoclasts or monocytes [12] and human bone marrow-derived mesenchymal stem cells [13] and human osteoblasts (hFOB 1.19) [14]. Additionally, studies on murine macrophage cells (RAW 264.7) [10,12], studies on postmenopausal women [15] and comparative studies of men and women to study the effect of certain treatments on the prevalence of fractures [16] have been included. For secondary osteoporosis models, researchers present models with female rats with glucocorticoid-induced bone loss [17], mice with dexamethasone-induced secondary osteoporosis [18] and rats with diabetes-induced osteoporosis [19].

## 2. Pathophysiology

Osteoporosis can be divided into primary and secondary types. Primary osteoporosis is usually a consequence of normal aging and is further divided into postmenopausal osteoporosis, age-related (senile) osteoporosis and idiopathic osteoporosis [20]. Osteoporosis caused by other diseases, disorders or other medication is referred to as secondary osteoporosis [20,21].

### 2.1. Primary Osteoporosis

#### 2.1.1. Postmenopausal Osteoporosis

Approximately 40–50% of women over 60 years old have osteoporosis [1]. Estrogen plays a key role in normal bone health by increasing calcium resorption and inhibiting bone resorption and calcium excretion. During menopause, the decrease in estrogen levels leads to osteoclast activation, causing an increase in bone resorption and thereby exceeding the ability of osteoblasts to form new bone, resulting in accelerated bone loss [22,23].

#### 2.1.2. Senile Osteoporosis

Senile osteoporosis occurs in both men and women and is associated with age. It is caused by a reduced bone formation [22], which might be partly explained by the loss of muscle mass occurring during aging [24]. Males usually exhibit a slower and more gradual loss in bone mass density (BMD) than women [25]. Both estrogen and testosterone have bone health-promoting effects [23], and deficiencies of these hormones due to aging might contribute to the disease [22,25]. Furthermore, other causes of age-related osteoporosis might be related to decreased vitamin D production and changes in the cell microenvironment, leading to changes in osteoblast function [25].

#### 2.1.3. Idiopathic Osteoporosis

Idiopathic osteoporosis is a rare disease that refers to the development of osteopenia and fractures with minimal or no trauma in young, healthy individuals without other identifiable secondary causes of osteoporosis [26]. This involves primary bone demineralization in a prepubertal onset (regardless of sex) and continues throughout puberty. Although its pathogenesis is unknown, it has been related to a very low rate of bone formation and a significant decrease in cancellous bone. The main symptoms are long bone fractures, back pain and difficulty walking or the inability to walk [27].

Idiopathic osteoporosis affects both sexes equally, and patients have been reported to present hypercalciuria, abnormal vitamin D functioning and insulin-like growth factor 1 and IL-1 production [26]. This abnormal functioning of vitamin D could be related to changes in the vitamin D binding protein (VDBP) and its polymorphisms, which affect the main transport of 25-hydroxyvitamin D (25(OH)D) in the circulation. Under normal conditions, it plays a role in maintaining stable levels during times of decreased 25(OH) availability and regulates the delivery of 25(OH)D to target tissues, altering affinity, activity and concentration [28]. Polymorphisms can change due to a single nucleotide polymorphism (SNP) in exon 11: rs7041 and rs4588 or in its glycosylation pattern. Furthermore, it has been shown that low serum VDBP levels correlate with low BMD, and therefore, VDBP could be a potential non-invasive biomarker for early osteoporosis detection. On the other hand, the SNP rs7041 has been found to have a higher frequency in osteoporosis [29].

### 2.2. Secondary Osteoporosis

Secondary osteoporosis occurs in almost two-thirds of men, more than 50% of premenopausal women and about 30% of postmenopausal women [21]. Secondary osteoporosis is characterized by low BMD or an increased risk of fractures caused by factors other than sex and age [21]. These factors are associated with lifestyles and habits, such as the consumption of tobacco, alcohol, diet, physical inactivity, underlying diseases and medications [21], as there are a wide range of diseases, treatments and drugs that can affect bone quality in both men and women of all ages. In men, the most common cause of secondary osteoporosis is treatment with exogenous glucocorticoids [25].

### 2.3. Other Types of Osteoporosis

#### Pregnancy and Lactation

During pregnancy, and especially during lactation, reductions in BMD occur as calcium is released from the skeleton of the mother to meet fetal demands. These changes are generally temporary and reversible, and most women do not suffer any clinically apparent changes in bone health [30]. However, when the body is unable to produce an adequate balance of compensatory mechanisms, pregnancy can lead to calcium metabolism-related diseases and decreased bone mass [31,32,33]. Hence, pregnancy and lactation-associated fractures are rare but may arise from other secondary causes of osteoporosis [34].

## 3. Bone Regulation

The study of the mechanisms of bone regulation will help us understand the processes by which osteoporosis is produced and to know which targets we can act on. Thus, in the following section, we will briefly comment on the most relevant mechanisms involved in bone formation and resorption.

### 3.1. Mechanical Factors

The adaptation and plasticity of our organs and tissues are key phenomena for the survival of living beings [35]. Therefore, the load supported by certain bones has a great influence on bone remodeling. That is how moderate and even intense physical activity, depending on the capacity of each individual, will play a fundamental role in the health of our bones, increasing their strength and density [36]. Through mechanical loading, exercise encourages mesenchymal stem cells to differentiate into osteoblast lineages, thereby producing more healthy bone cells [37] as a part of an adaptation mechanism. This is why it is important to consider physical activity as playing a key role in maintaining a healthy skeleton, including strength exercise and physical activity in all age ranges, starting before, during and after puberty. Physical exercise provides positive effects on the amount of bone mass accumulated in adulthood, reducing the relative risk of fractures, and as it becomes a consistent habit, it will have substantial implications in the prevention of osteoporosis [38].

Mechanical forces guide tissue morphogenesis, including processes such as cell migration, tissue folding and organ formation. This is a process known today as mechanotransduction, in which stimuli from the extracellular matrix are converted into biochemical signals within the cell, with consequences on cellular structure, genetic expression and physiological functions. Osteoblasts, osteoclasts, osteocytes, T cells, B cells, megakaryocytes and lining cells are potentially mechanosensitive and interrelated, as they all respond to mechanical loading [39].

**Figure 1:**
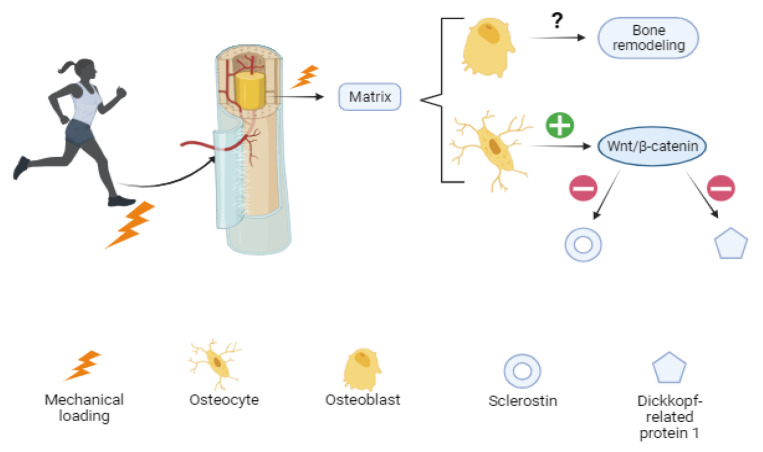
Effects of mechanical loading on bone matrix cells and its impact on the regulation of signaling pathways (Wn/β-catenin) that determine the lower coding of genes responsible for the reduction of bone mass (SOST and Dkk1). ?—It has been proposed that osteoblasts alter bone remodeling; however, the mechanisms are not yet clear.

Although “mechanobiology” is a recent interest of researchers and the mechanisms are not yet completely clear, it has been proposed that osteoblasts, the cells that produce bone matrix, translate mechanical loading into biochemical signals that affect bone modeling and remodeling. Osteocytes in the mineral matrix seem to respond by upregulating the Wnt/β-catenin signaling pathway, which is suggested to be responsible for decreasing the expression of negative regulators of the pathway such as sclerostin (codified by SOST gen) and Dickkopf-related protein 1 (Dkk-1) [39]. Increased bone mass has been reported in clinical trials using antibodies against SOST, suggesting this could be a potential target for the treatment of bone-related diseases such as osteoporosis [40] (Figure 1).

In addition to these mechanical factors, there is also an endocrine relationship through muscle and bone interactions. Bones were not recognized as endocrine organs until 2007 by Lee and his collaborators, and their relationship with muscle tissue is understood through “bone-muscle crosstalk” [41]. Within this crosstalk, irisin, a polypeptide that comprises 112 amino acids, is a messenger derived from skeletal muscle during exercise to regulate metabolism. It is the most studied myokine in this area, as serum irisin levels have been positively associated with bone mineral status, whereas low irisin levels are associated with an increased risk of hip fracture [41]. This cross-talk would allow interactions between irisin levels and the activity of osteoclasts, osteoblasts and bone mesenchymal stem cells (BMSCs), as well as in an autocrine manner in the muscle, giving rise to a better understanding of the concept of “osteosarcopenia”, used to define a synergistic condition of low bone mineral density with muscle atrophy and hypofunction [41].

### 3.2. Hormonal Factors

#### 3.2.1. Calcemia-Regulating Hormones

Our endocrine system has a key role in bone remodeling since bones constitute a store of calcium and phosphate for the control of plasma levels of these elements [42]. This involves hormones such as parathormone (PTH), calcitriol (1,25 dihydroxycholecalciferol) or calcitonin. The first two have the function of increasing calcium levels in the blood, and calcitonin decreases them [43,44]. However, its activity can vary, as it is subject to its secretion levels, and the interaction between them will determine bone formation or degradation [44].

PTH stimulates the activity of osteoclasts to increase Ca^2+^ levels in the blood in situations of hypocalcemia in a physiological way [45]. However, it has been proven that if administered intermittently, it also stimulates bone formation [43].

The effect of calcitriol, or the active form of vitamin D, has actions on multiple levels. It increases the intestinal absorption of calcium, inhibits the secretion of PTH by acting on the parathyroid glands and also intervenes in bone mineralization [46]. It has been studied that calcitriol at high concentrations is not useful for increasing bone density or bone strength. On the contrary, bone density decreased [47], because calcitriol increases osteoclast production, which increases bone resorption.

Calcitonin mediates the phenotypic change of osteoclasts and, therefore, their activity. This produces the inhibition of the secretion of proteolytic enzymes from osteoclasts, stimulating bone deposition [44]. In fact, it has been used to treat bone disorders such as osteoporosis, hypercalcemia and Paget’s disease [48].

#### 3.2.2. Glucocorticoids

Cortisol is a steroid hormone secreted by the adrenal gland. It is released by a physiological response to stress and low glucose levels. This hormone also influences bone cells, depending on the dose. It is essential for the maturation of osteoblasts by promoting their differentiation from mesenchymal progenitors, but it can also decrease their activity. In fact, the prolonged use of corticosteroids decreases bone formation, predominantly affecting the trabecular bone, with special repercussions at the vertebral level [49].

#### 3.2.3. Sex Hormones

Sex hormones, such as testosterone or estradiol, exert an anabolic effect on bone mass since they inhibit resorption and stimulate bone formation. The antiresorptive effect of estrogen on the blockage of osteoclasts is more powerful than the effect of testosterone. However, the latter exerts a special influence on mature osteoblasts and osteocytes, stimulating bone formation [50].

Estrogens decrease the responsiveness of osteoclast progenitor cells through the transcription of the receptor activator of nuclear factor kappa B ligand (RANKL), which reduces its useful life, stimulates the proliferation of osteoblasts and decreases its apoptosis [51]. The mechanism is driven by the activation of transforming growth factor beta (TGF-β) mediated by osteoblasts in which the inhibition of interleukin 6 (IL-6) production, a key stimulus for resorption, is also involved [52]. It is known that estrogen deficiency also increases the apoptosis of osteocytes, which alters the mechanosensory function of the canalicular microdamage repair system, contributing to bone frailty [50].

On the other hand, androgens act by increasing bone mass and stimulating its formation in both men and women. Most studies carried out in vitro demonstrate a stimulating effect on osteoblastic proliferation and differentiation while inhibiting their apoptosis [53]. The effector pathway of these effects has been proposed to be a stimulation of TGF-β and insulin-like growth factor 1 (IGF-1) together with an inhibition of IL-6 [53].

**Figure 2:**
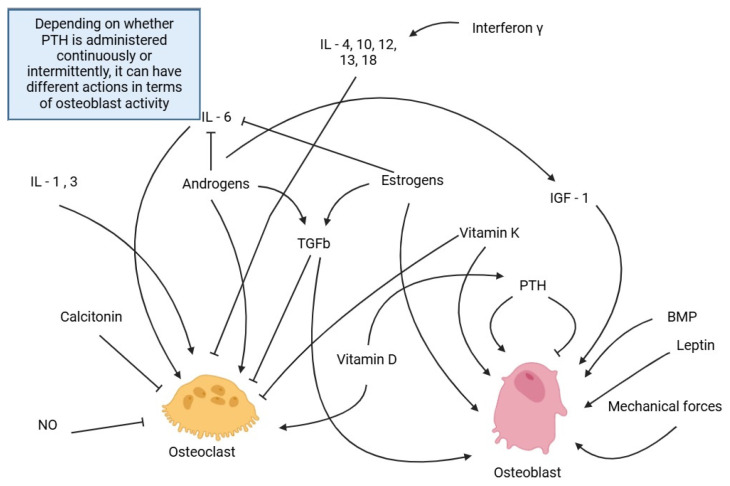
Hormonal and mechanical factors on osteoblast and osteoclast formation.

Figure 2 summarizes the hormonal and mechanical factors have on osteoblast and osteoclast formation.

#### 3.2.4. Growth Hormones

Growth hormones (GH) have a special influence on bone growth and maturation during body development. IGF-1, secreted by the liver through the action of this hormone, stimulates the replication of osteoblastic precursors. Specifically, it activates their function in the synthesis of matrix and collagen, in addition to inhibiting their apoptosis [54]. However, the different isoforms have different functions. For example, insulin-like growth factor binding protein 4 (IGF-BP4) has an inhibitory role in the replication and differentiation of osteoblasts, while insulin-like growth factor binding protein 5 (IGF-BP5) would be a stimulator [54]. On the other hand, osteoblasts also have the ability to synthesize IGF 1 and 2, stimulated by hormones such as PTH, exerting on the bone not only cell proliferation but also functions such as collagen production and matrix apposition [55].

After the third decade of life, there is a progressive decrease in GH secretion of approximately 15% for each decade of adult life [56]. During a 10-year follow-up study, the greatest increase in BMD was achieved in year six at the lumbar spine (+6%) and total hip (+13%). They showed a sustained positive effect of GH replacement therapy on bone density, in subjects with adult GH deficiency, with no effect on trabecular bone score, as an indirect measure of trabecular bone microarchitecture [57].

### 3.3. Other Biochemical Factors

#### 3.3.1. Interleukins

Interleukins exert diverse and sometimes contrary effects on bone. Interleukin 1 (IL-1) is an activating factor of mature osteoclasts promoting bone resorption [58], IL-3 stimulates the differentiation of precursors into mature osteoclasts and IL-11 stimulates the maturation of osteoclast precursors, thus producing an increase in erosive activity in bone. These factors are mainly involved in situations of stress and result in an increase in proinflammatory cytokines. However, other interleukins such as IL-4, 13 and 10 inhibit osteoclastic maturation [59], IL-6 decreases osteoclast migration and differentiation [60] and IL-12 and 18 inhibit osteoclast differentiation [61].

#### 3.3.2. TGF-β

Another factor, such as TGF-β, promotes bone formation by stimulating the synthesis of osteoprogeterin (OPG), a key protein in bone formation, which is why it is considered a crucial signal between the cells that maintain bone remodeling and plays an important role in fracture repair [62]. Bone morphogenic proteins (BMPs) are peptides of the TGF-β family, whose action on specific receptors stimulates osteoblastic differentiation and induces bone formation during skeletal development. In adult tissue, their function is to maintain a continuous supply of osteoblasts, although, as we have mentioned before with other factors, it has been shown that they can also stimulate bone resorption and induce the differentiation of immature mesenchymal progenitor cells into osteoblasts [63].

#### 3.3.3. Interferon Gamma

Interferon gamma (INF-ɣ) exerts a potent inhibition of osteoclast formation while stimulating the synthesis of various cytokines, including IL-18 [64]. The INF-ɣ action is produced mainly by T lymphocytes and natural killer (NK) cells, causing the activation of the inflammatory process and therefore intervening in immune responses in addition to its activity at the bone level [65].

#### 3.3.4. Nitric Oxide

Nitric oxide is a molecule that plays several roles within the physiology of the body, among which the most notable is its great vasodilating action. It also acts as a powerful neurotransmitter. The main sources of nitric oxide in the bone are osteoblasts and endothelial cells, which have gained importance in recent years as regulators of remodeling since they are mediators of the anabolic effect of estrogens [66] the mechanical response of the bone and exert osteoclastic inhibitory effects [67].

#### 3.3.5. Leptin

Leptin is a hormone that is released from adipose tissue and has anorexigenic and thermogenic activity, thereby promoting fat consumption. Its effect on the skeleton has been highlighted through its receptors in mesenchymal, preosteoblastic cells and mature osteoblasts, in addition to its anabolic effects, through which it promotes osteoblastic differentiation, thus increasing the synthesis of matrix proteins and reducing the apoptosis of the osteoblasts [68]. Additionally, it has been described that osteoclastic function also decreases due to an increase in OPG and a decrease in RANKL [69]. A negative correlation between leptin and remodeling markers has been found in women with postmenopausal osteoporosis and a positive correlation with bone mass [70].

#### 3.3.6. Vitamin K

Vit K (VK) limits osteoclastogenesis by promoting the transition from osteoblast to osteocyte. This effect occurs mainly through proteins such as osteoclacin and matrix protein Gla (MGP protein). The effect of VK on bone health and remodeling also involves MGP, which promotes bone formation by positively regulating Wnt/β-catenin signaling but also exerts an inhibitory effect on bone mineralization. At the late stage of osteoclast differentiation, MGP is highly expressed, delineating a negative feedback loop to achieve tight control of osteoclast formation. VK regulates osteoblastogenesis and osteoclastogenesis through the nuclear factor κB (NF-κB) signal transduction pathway. NF-κB signaling exerts two functions: on the one hand, it stimulates the development and resorption of osteoclasts, while on the other hand, it inhibits the differentiation and activity of osteoblasts. VK2 prevents NF-κB activation independently of γ-carboxylation, leading to bone formation and reduced bone resorption [71].

#### 3.3.7. Mechanisms of Bone Remodeling Action: OPG/RANKL/RANK System

Regarding the local regulation of bone cell function, after the discovery of the OPG/RANKL/RANK system, there is a clearer picture of bone remodeling in general. The studies in mice that helped us understand the function of the OPG-RANKL system were published many years ago. OPG knockout mice develop severe osteoporosis; however, the overexpression of OPG in transgenic mice produces osteopetrosis, a group of rare disorders that cause bones to grow abnormally and become too dense, making them brittle, which causes them to fracture easily. This is produced by the inhibition of the osteoclast maturation process, preventing the renewal of the bone matrix and resulting in an aged matrix [72].

The absence of RANKL in mice also produces osteoporosis, the alteration of the dentition due to the absence of mature osteoclasts, the absence of lymph nodes and the deficiency of B and T lymphocytes. On the other hand, the administration of RANKL in its soluble form produces an increase in the activity of osteoclasts, osteoporosis and hypercalcemia [73]. It can be concluded that the expression of RANKL favors the maturation of osteoclast precursors; the same scenario occurs in OPG knockout mice, triggering the appearance of osteoporosis.

**Figure 3:**
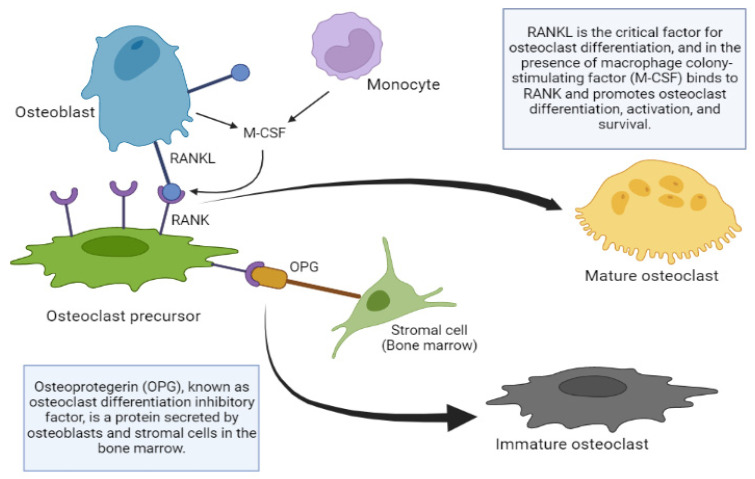
Action mechanism of the OPG/RANKL/RANK system in osteoclast activation and differentiation.

RANKL is the critical factor for osteoclast differentiation, and in the presence of macrophage colony-stimulating factor (M-CSF), it binds to RANK and promotes the differentiation, activation and survival of osteoclasts, as well as their adherence to the bone surface [74]. Other molecules of the tumor necrosis factor (TNF) family such as TNF-α, also intervene in this differentiation process, possibly favoring the action of RANKL or stimulating similar intracellular pathways. Osteoblastic cells can also produce M-CSF, which facilitates the replication of osteoclastic precursors and can induce the expression of RANK in these precursors [75]. OPG binds to the RANKL, neutralizing its action and thus inhibiting osteoclastogenesis [76]. The signaling cascade that occurs after the union of RANKL and RANK is not completely known, although it is known that factors associated with the TNF receptor and especially IL-6 are involved. Mature preosteoblasts and osteoblasts express RANKL, which binds to RANK expressed on the surface of osteoclast precursors and favors the differentiation of precursors towards the formation of mature osteoclasts [73]. OPG binds to and neutralizes RANKL and, as a result, inhibits osteoclastogenesis and osteoclastic activity while inducing osteoclast apoptosis [77]. The production of OPG relative to that of RANKL increases with osteoblast differentiation, allowing the mature osteoclast to fill the remodeling space (Figure 3).

The regulation of the remodeling process integrates the union of multiple factors of diverse origin, which gives great complexity to this process.

## 4. Risk Factors

Until now, we have described osteoporosis as a chronic disease characterized by an alteration of the microstructure of the bones that makes them less dense and therefore more fragile and susceptible to fractures. It is also the most serious complication and cause of disability and death in older adults, especially women.

It has many causes, including a drop in estrogen levels in women or testosterone in men, unhealthy lifestyles, hormonal and nutritional disorders and genetic disorders, and it can even be a secondary consequence of the use of medicines. These triggers have direct and indirect effects on bone tissue cells, generating an imbalance either in favor of the activation of osteoclasts or the inhibition of osteoblasts, ultimately reducing the quality of the bone matrix.

Osteoporosis risk factors can be divided into modifiable and non-modifiable. The modifiable factors include body weight, smoking, alcohol consumption, physical inactivity, dietary calcium deficiency and the long-term use of glucocorticoids. The non-modifiable factors include sex, age, race and genetic characteristics.

Below, we will expand on the potential risk factors around this disease.

### 4.1. Hormones

In general, the decrease in some hormones during aging affects the formation and maintenance of bones.

17β-estradiol or E2, as previously mentioned, exerts a potent effect on osteoclast inhibition, thereby preventing osteoporosis. During menopause and especially in postmenopausal women, the decrease in and loss of E2 implies a greater predisposition to a loss of bone mass. The presence of alpha and beta receptors on human osteoblasts, osteocytes and osteoclasts indicates the influence of these hormones on bone remodeling. However, receptors have only been identified on osteoblasts and osteocytes. In addition, estrogen can increase the levels of (PTH) and calcitriol (1,25(OH)2-vitamin D3), which stimulate intestinal calcium absorption and its reabsorption in the renal tubule. PTH can also act by activating bone resorption by regulating the activity and number of osteoclasts. GH stimulates bone resorption by interacting with the GH receptors present in osteoblasts [78].

Glucocorticoids, which are widely used in inflammatory or immune system disorders in high doses (2.5 to 7.5 mg/day of prednisone) and with prolonged use, generate a side effect known as “glucocorticoid-induced osteoporosis” in a significant number of patients (30–50%), especially during the first year of treatment [79]. This occurs because glucocorticoids increase bone resorption by stimulating the differentiation and maturation of osteoclasts. They also inhibit osteoblastogenesis and promote the apoptosis of osteoblasts and osteocytes, which ends in a decrease in bone formation, and suppress the production of IGF-1 or GH [80].

### 4.2. Genetics

The onset of osteoporosis is affected by peak bone mass, which is mainly genetically determined [22]. Genetic variants related to bone mass include low-density lipoprotein receptor-related protein 5 (LRP5), OPG, sclerostin (SOST) and estrogen receptor 1. Additionally, genes of the RANK pathway have been associated with bone mass [22]. Furthermore, the vitamin D receptor (VDR) gene regulates the transcription of several bone homeostasis-related genes. Allelic variations of the VDR gene have been linked with greater incidences of osteoporotic fractures [81].

### 4.3. Physical Inactivity

A lack of physical activity has been associated with alterations in bone remodeling homeostasis. Interestingly, the loss of muscle mass due to aging and inactivity appears to be linked with osteoporosis. For example, muscle unloading decreases integrin expression and alters the Sclerostin-LRP5/6-receptor-regulated Wnt/β signaling pathway and IGF, BMP and PTH levels [24].

It is widely known that a lack of activity can induce bone loss and muscle atrophy. In terms of loading, the effects of microgravity on bone tissue in missions from 4.5 to 14.5 months demonstrated that bone mineral density and mineral content decrease throughout the bodies of the astronauts, mainly in the lower body (pelvis and femoral neck), and there is a loss of bone minerals in the lower appendicular skeleton at a rate of close to 2% per month, accompanied by muscle atrophy. However, the mechanism of these phenomena is still unclear. In summary, the absence of functional loading, due to inactivity, results in a loss of bone mass, while exercise or increased activity results in an increase in bone mass [39].

### 4.4. Nutrition

Nutrition plays an important role in the prevention and risks of osteoporosis, and it is worth highlighting the special influence of the following compounds.

#### 4.4.1. Vitamin D

Vitamin D is crucial for maintaining bone homeostasis, and low vitamin D serum levels are associated with osteoporosis. Vitamin D affects several metabolic processes in the bone, including the regulation of serum calcium, phosphorus levels and PTH excretion and the modulation of bone remodeling by osteoblasts and osteoclasts [1].

#### 4.4.2. Calcium

Calcium is the main bone-forming mineral [24]. Low dietary calcium intake might cause a higher risk of osteoporotic fractures [2]. When the dietary intake of calcium is low, a sufficient serum calcium level is achieved by inhibiting bone mineralization while simultaneously increasing bone resorption, leading to a loss of BMD. Therefore, a sufficient dietary intake of calcium is necessary for bone homeostasis [1]. The efficiency of calcium absorption is highly variable between individuals. Calcium is absorbed in the intestine through two mechanisms: active and passive absorption. Active absorption in the proximal small intestine is regulated by dietary intake and bodily needs, which is controlled by 1,25(OH)2D, but this active transport is saturable due to the requirements of calcium-binding proteins for the transport of calcium across the intestinal cells into the blood. Therefore, during the intake of large amounts of calcium at once, only a certain amount will be absorbed via active absorption. On the other hand, passive absorption, which occurs in the distal regions of the intestine, does not saturate and increases with the increased dietary intake of calcium. Other compounds of the diet that make calcium more soluble, such as milk proteins and lactose, help stimulate the passive absorption of calcium [82].

Estrogen deficiency causes decreased absorption and thereby intestinal resistance to the active form of vitamin D, which allows the body to absorb calcium. Adequate levels of vitamin D are essential for calcium absorption [82]. Some compounds of the diet, such as cellulose, phosphate, oxalate and methoxylated pectins can form insoluble complexes with calcium, thereby reducing its absorption [83].

Calcium and magnesium compete for absorption, and therefore, excess calcium not only blocks the entry of magnesium but also increases the excretion of magnesium through the kidneys. There is an optimal ratio for dietary calcium in relation to magnesium intake to optimize bone health. Calcium intake that is below 2.2 times or exceeds 3.2 times that of magnesium is associated with poorer bone outcomes, and thus, balanced intakes of both dietary calcium and magnesium are important for maintaining BMD and preventing osteoporosis [84].

#### 4.4.3. Magnesium

Magnesium is involved in bone crystallization, stabilization and ATP metabolism [2,24]. Magnesium deficiency has been shown to decrease BMD by altering the homeostasis of vitamin D and PTH levels and by decreasing osteoblastic activity [85]. Inadequate magnesium intake is also associated with low-grade inflammation, which can stimulate adverse effects on bone remodeling. Additionally, low levels of magnesium have been shown to increase the size of hydroxyapatite crystals in the bone, leading to decreased bone quality [24,86].

#### 4.4.4. Fluoride

Fluoride has been shown to affect bone health in both high and low concentrations. A high concentration of fluoride stimulates osteoblasts, but it also increases the size of bone crystals, possibly making the bone more prone to fractures. However, inadequate fluoride concentrations appear to increase BMD and decrease the risk of fractures [87].

#### 4.4.5. Protein

Dietary protein intake is vital to the production of hormones and growth factors regulating bone metabolism [87] and signaling [24]. Furthermore, protein is involved in the collagen structures of the bone matrix, and dietary protein has been shown to increase IGF-1, which promotes bone formation [87].

#### 4.4.6. Other Dietary Compounds

Several other minerals, such as zinc, phosphorus and manganese, have also been identified to affect bone health. For example, these minerals are required for collagen synthesis, bone mineralization and matrix formation. Additionally, alongside vitamin D, several other vitamins, such as vitamins A, B, C and K, influence bone homeostasis [24,87].

## 5. Treatment and Prevention

As has been mentioned, osteoporosis is a disease with an increasing prevalence in men and women since life expectancy has increased. Therefore, the two objectives that are considered when treating this disease are achieving a long-term reduction in fractures (anti-fracture treatments) and safety. Fractures can be prevented or treated with drugs that, on occasion, can have anti-bone remodeling functions. Below, we present some of the most-used treatments and dietary compounds with preventative effects. See Appendix A for research methodology.

### 5.1. Pharmacotherapy

#### 5.1.1. Antiresorptive Drugs

Biphosphonates are one of the most widely used antiresorptive drugs [88]. Their action is based on decreasing the rate of bone resorption. Another antiresorptive treatment includes a fully human monoclonal antibody known as denosumab (DMab) and selective estrogen modulators (SERMs). DMab inhibits RANKL, which is essential for osteoclastic activity. SERMs, such as raloxifene, act as agonists on estrogenic receptors of the bone, thus inhibiting bone resorption [89]. Currently, we can find different treatments with bisphosphonates on the market such as Ibandronate, Alendronate or Zoledronic acid [89].

Furthermore, hormonal treatments, including estrogen and testosterone therapies, might be useful for the prevention and treatment of osteoporosis. Estrogen and testosterone are significant inhibitors of bone resorption [89], and hormone replacement therapy or a synthetic steroid called tibolone can increase BMD [81]. Another hormonal treatment option can be calcitonin, which also inhibits osteoclastic activity [90].

#### 5.1.2. Anabolic Treatments

Anabolic treatments include the parathyroid hormone analog teriparatide. Intermittent small doses of teriparatide increase bone formation more than resorption, leading to a total increase in BMD [81,89]. Teriparatide binds through the N-terminal moiety to PTH type 1 receptors, which are G-protein coupled receptors (GPCRs) expressed on surfaces of various bone cells, including osteoblasts and osteocytes. Although PTH activates protein kinase A (PKA) and also protein kinase C (PKC) dependent signaling pathways, the PKA-dependent pathway is the most used for its anabolic effects on bone [91].

Another pharmacological treatment is romosozumab, which stimulates bone formation while inhibiting bone resorption [81]. Romosozumab is a monoclonal antibody targeting sclerostin, the Wnt/β-catenin antagonist, a protein that interferes with osteoblastic maturation [81]. This inhibits sclerostin’s ability to bind to the LRP5 and LRP6 receptor proteins, allowing the Wnt/β-catenin pathway to occur, thus promoting the increasing of cortical and trabecular bone mass by dual effects: a) an increase in osteoblastic activity, thereby increasing bone formation, and b) a decrease in osteoclastic activity, thereby decreasing bone resorption [92].

On the other hand, there is strontium ranelate (SR), mostly used in postmenopausal women, which is a compound that has a double mechanism of action: it stimulates the formation of bone tissue and decreases bone resorption. SR shows physical and chemical similarities with calcium; which is why SR acts through the calcium-sensing receptor (CaSR) in bone tissue cells [93].

### 5.2. Dietary Compounds

Dietary compounds may have preventive effects for the development of the disease or even effects that contribute to the reversal of the disease when it is already developed. In particular, dietary polyphenols have an influence on the preventive or adjuvant level against diseases (both positive and negative), including osteoporosis. Next, we will discuss some of the most important polyphenols in this regard, as well as other dietary compounds.

#### 5.2.1. Caffeic Acid

Caffeic acid is a metabolite of hydroxycinnamate and phenylpropanoid commonly synthesized by several plant species, present in coffee, tea, wine, blueberries, apples and honey [94].

The studies relating caffeic acid to osteoporosis are scarce. However, in a recent study, the effects of caffeic acid were investigated on osteoblasts and osteoclasts in vitro using mice bone marrow-derived mesenchymal cells [11]. They found that caffeic acid at 5 and 10 µM regulated osteoblast and osteoclast cell proliferation, increasing and decreasing them, respectively, and accelerated bone mineralization. Additionally, they found that it upregulated osteopontin (OPN), osteocalcin (OCN) and bone morphogenic proteins, as well as matrix metalloprotease-2 (MMP-2) and cathepsin-K proteolytic markers in osteoclast cells [11].

Tolba et al. studied the effects of caffeic acid phenethyl ester (CAPE), a derivative of caffeic acid, in glucocorticoid-induced osteoporosis, reporting that CAPE opposed dexamethasone (DEX)-mediated alterations in bone histology and tartrate-resistant acid phosphatase (TRAP) activity [95]. They also found that CAPE reduced caspase-3 activity in femur tissues and restored oxidative balance and runt-related transcription factor 2 (RunX2) expression. Finally, the co-administration of CAPE with DEX normalized RANKL/OPG ratio and Akt activation indicated a reduction in DEX-osteoclastogenesis [95].

Another study examined the effects of caffeic acid 3,4-dihydroxy-phenethyl ester (CADPE) (another derivative of caffeic acid) on osteoclastogenesis by treating mouse bone marrow monocytes (BMMs) and RAW 264.7 cells with dosages between 0.1 and 5 µM [12]. It demonstrated that CADPE suppresses osteoclastogenesis and bone loss by inhibiting RANKL-induced MAPKs and Ca^2+^-NFATc1 signaling pathways in a dose-dependent manner [12].

#### 5.2.2. Resveratrol

Resveratrol is a well-known polyphenol with a stilbene structure that can be found in grapes, wine, blueberries, blackberries and peanuts [96].

Recently, a 24-month randomized, double-blind, placebo-controlled, two-period crossover intervention called “The Resveratrol for Healthy Aging in Women (RESHAW)” was conducted to evaluate the effects of 75 mg resveratrol twice daily in postmenopausal women from 45 to 85 years old [15]. After 12 months of supplementation, they reported positive effects on bone density in the lumbar spine and the neck of the femur, such as a 7.24% reduction in the C-terminal telopeptide type-1 collagen level, which is a bone resorption marker, compared with the placebo. An increase in bone mineral density was found in the femoral neck, which resulted in a reduction in the 10-year probability of major and hip fracture risk [15]. 

In an in vivo study on ovariectomized rats, Khera et al. found that 625 µg/kg/day resveratrol restored the RANKL/OPG ratio, slightly increased BMD and significantly reduced IL-23, IL-17A, IL-1β and TNF-α cytokine expression levels after 4 weeks of treatment [9]. In another study performed in vivo and in vitro in ovariectomized rats and RAW 264.7 cells, Feng et al. explored the mechanisms by which resveratrol prevents bone loss through its antioxidant effects [10]. They found that the administration of 40 mg/kg resveratrol once daily for 10 weeks results in elevated OPG, attenuated bone microarchitecture damage and decreased RANKL, thereby inhibiting osteoclastogenesis in the ovariectomized rats. In vitro, they found the improved oxidative stress status of the cells and the decreased mRNA expression of osteoclast-specific enzymes through the inhibition of the PI3K/AKT signaling pathway after 10 µM treatment for 24, 48 and 72 h [10].

Song et al. published studies of the effects of resveratrol on human bone marrow-derived mesenchymal stem cells as well as in ovariectomized rats. They found that miR-193a was overexpressed and SIRT7 downregulated in osteoporosis [13]. However, the in vitro treatment of 10 μM resveratrol suppressed miR-193a to promote osteogenic differentiation through SIRT7 upregulation. Furthermore, after 8 weeks of oral administration of 50 mg/kg resveratrol, they found that resveratrol exerted beneficial effects through miR-193a/SIRT7-mediated NF-kB to alleviate osteoporosis in vivo [13].

#### 5.2.3. Tangeretin

Tangeretin belongs to a group of polymethoxylated flavones found extensively in citrus fruits [97].

It has previously been described that the silencing of miR-137 enhances osteoblastic differentiation potential through the coordination of lysine-specific histone demethylase 1 (LSD1) and bone morphogenetic protein 2 (BMP2) [98]. An in vitro and in vivo study by Kang et al. showed that the silencing of miR-137-3p significantly improves osteogenesis by enhancing the expression of RunX2. This means an increase in the serum content of stromal-derived factor 1 alpha (SDF-1α), also known as CXC motif chemokine 12 (CXCL12), led to an increase in the number of endothelial progenitor cells through the up-regulation of CXCL12 [99].

In a study with human adipose-derived stem cells, treatment with 5 μM of tangeretin reversed the effects of miR-137 knockdown on osteogenic promotion. They concluded that a NOTCH1/LSD1/BMP2 co-regulatory signaling network could explain the modulation of miR-137 on osteoblast differentiation, providing a mechanism-based rationale for the miRNA-targeted therapy of bone defects [98].

In another in vitro study on RAW 264.7, macrophage treatment with 30 µM of tangeretin for 5 days suppressed lipopolysaccharide-induced osteoclast formation and bone resorption factors associated with inflammation. Additionally, they found that tangeretin suppressed the receptor activator of NFκB ligand-induced osteoclastogenesis [100].

#### 5.2.4. Urolithins

Urolithins are the intestinal microbial metabolites of ellagic acid and ellagitannins present in pomegranates, berries and walnuts [101,102]. The bacteria *Gordonibacter urolithinfaciens* and *Gordonibacter pamelaeae* play an important role in the conversation of urolithin; however, the microorganisms responsible for the complete transformation into the final urolithins are still unknown [103].

Tao et al. studied the effects of urolithin A in ovariectomized mice and macrophages. The in vivo results indicated that the oral administration of 10 mg/kg/day and 20 mg/kg/day of urolithin A for 8 weeks effectively reduced ovariectomy-induced systemic bone loss. In vitro, urolithin A modulated macrophage polarization and alleviated inflammatory response. Additionally, the attenuated expression of inflammatory factors prevented nuclear translocation of NF-κB and suppressed RANKL-triggered osteoclastogenesis [104].

Macrophage polarization refers to the fact that macrophages adopt different functional programs in response to their microenvironment. They can be subdivided phenotypically as M1 and M2. The M1 subtype is described as pro-inflammatory (direct defense against pathogens, phagocytosis and secretion of pro-inflammatory cytokines), and the M2 subtype is the opposite, regulating the termination of inflammation and repair of damaged tissues (anti-inflammatory). Urolithin could mediate the polarization of macrophages towards the M2 subtype, both decreasing inflammation and facilitating regeneration. It has been described how this polarization of macrophages could contribute to osteoblastic differentiation, increased osteogenesis and bone mineralization. The modulation of the macrophage subtype could be an objective when treating osteoporosis [105].

In another study by Tao et al., urolithin A was found to inhibit RANKL-induced osteoclastogenesis in bone marrow macrophages and reduce the expression of osteoclast-related genes and bone resorption in vitro after treatments of 5 and 10 µM for 24 and 48 h [106]. They also reported that the intragastric administration of 10 and 20 mg/kg urolithin A four times a week for 20 weeks inhibited bone loss in aging mice [104].

In an in vitro study, RAW 264.7 cells treated for 48 h with 25 μM were found to have repressed RANKL-induced osteoclast differentiation through the regulation of Akt1, p38 and ERK1/2 signaling [101]. Although urolithin A is known to be the most potent metabolite of ellagitannins and ellagic acid, urolithin B has been shown to exert positive effects as well. In a recent study, Li et al. treated ovariectomized mice intraperitoneally with 10 and 50 mg/kg of urolithin B every two days for eight weeks, while BMMs and RAW 264.7 cells were treated with 1, 5 and 25 µM for 12, 24 and 72 h [107]. They found the inhibition of osteoclast differentiation and the suppressed expression of the osteoclast-related genes MMP9, CTSK, NFATc1 and c-fos in vitro. Additionally, they reported that urolithin B suppressed phosphorylation and the degradation of IκB and suppressed the phosphorylation of P65 in the NF-κB pathway. In vivo, they found significant improvement in the distal femur and fewer MMP9 and TRAP-positive osteoclasts in bone tissues [107].

#### 5.2.5. Oleocanthal

Oleocanthal is a compound isolated from olive oil; it is derived from ligstroside and is the dialdehydic form of decarboxymethylenolic acid linked to tyrosol 1. It is also known that it has a homologous effect to the non-steroidal anti-inflammatory drug ibuprofen, which also has antioxidant properties that imply a benefit in the prevention of certain diseases such as Alzheimer’s [108]. Oleocanthal has been thoroughly researched for several health-beneficial properties, namely anti-inflammatory, antioxidant, antimicrobial, anti-cancer and neuroprotective. Most data on the health benefits of this compound are derived from cellular studies and animal studies [109]. According to some studies carried out in vitro, this compound already shows powerful anti-inflammatory effects at concentrations of 25 and 50 µM [110]. In other studies, it was observed that oleocanthal is capable of binding to estrogen receptors, which at the bone level implies an attenuation of bone mass loss, using rats with postmenopausal osteoporosis as study models [111].

Recent studies associate the consumption of oleocanthal with a decrease in inflammatory factors (IL-1, 3 and 6) involved in the activation of osteoclasts, activating bone resorption processes. For this reason, it could be determined that the consumption of oleocanthal prevents the loss of bone matrix [112].

#### 5.2.6. Naringenin

Naringenin is a citrus flavonoid found in grapefruit that has been shown to help with osteoporosis, cancer and cardiovascular disease due to its antioxidant and anti-inflammatory properties [113]. According to some studies carried out in vitro, naringenin can improve the synthesis of osteoprotegerin in addition to BMP in osteoblastic cells. It was also found that the administration of naringenin produced effects on osteocalcin levels, causing an increase in them [114]. Currently, in vivo studies have been described using ovariectomized female rats, to which a treatment of naringenin and another preparation of naringenin in nanosuspension were administered orally at a dose of 20 mg/kg of body weight. The results showed an improvement in bone density in women treated with naringenin nanosuspension, concluding that bioavailability can improve bone formation and therefore provide a solution to the problem of osteoporosis [115].

It has also been shown that naringenin treatments carried out in vitro with mouse cells at concentrations between 10 and 15 µM can stimulate the differentiation of BMSCs into osteoblasts. RANKL-induced osteoclastogenesis was also inhibited, reaching total suppression when naringenin was administered at a concentration of 50 µM [116]. Therefore, all these works demonstrate that the administration of naringenin protects the skeleton since it has an inhibitory effect on osteoclastogenesis while at the same time favoring osteoclastogenesis, all of which ultimately results in an improvement of the levels of the calcified matrix. It is true that there is a lack of clinical studies, which would be very interesting in order to validate these properties and finish determining the safety that this polyphenol may have [117].

#### 5.2.7. Curcumin

Curcumin is a bioactive compound that is extracted from the species Curcuma longa. It has been shown that this compound has various biological effects, among which its anti-inflammatory, antioxidant and anti-infective action can be highlighted [118]. Some studies have determined that the administration of curcumin is capable of reducing bone loss in ovariectomized rats, which were treated with curcumin with a dose of 110 mg per kilo of weight [119]. As we have mentioned, curcumin has an antioxidant action; this is reflected in studies where the activation of factor 2 related to glycogen synthase kinase 3β-nuclear factor erythroid 2, and the elimination of ROS would be essential to promote the survival and correct functionality of osteoblasts against the damage caused by oxidative stress. This effect was visible with treatment at a concentration of 5 µM [120]. Another study in which postmenopausal women with primary osteoporosis were administered a treatment consisting of capsules that contained 80 mg of curcumin demonstrated that the consumption of this substance activated some biomarkers of bone turnover. This study carried out in women also demonstrated the safety of using this compound as a treatment for osteoporosis [121]. Curcumin is also considered an activator of autophagy as well as an inhibitor of osteoclastogenesis since some studies have shown that it directly activated autophagy in osteoclast precursors, in turn inhibiting the stimulatory effect of RANKL [122].

#### 5.2.8. Ferulic Acid

Ferulic acid is a natural phenolic compound found in the cell walls of cereals, vegetables, fruits and nuts. Numerous studies have demonstrated that ferulic acid (FA) inhibits the formation and differentiation of osteoclasts and stimulates the differentiation and function of osteoblasts. Hou et al. demonstrated that treatment with 20 and 30 mg/kg body weight of ferulic acid in rats with glucocorticoid-induced osteoporosis increased bone mineral density. This effect was mediated by the increase in SIRT1 levels and the reduction of NF-κB [123]. In ovariectomized rats, the administration of 20 mg/kg/day for 12 weeks of FA increased bone density and mineral content, decreasing bone loss associated with osteoporosis. In vitro, treatment with 25, 50 or 100 μM of ferulic acid in RAW 264.7 murine macrophage cells decreased RANKL-induced osteoclastogenesis by downregulating the MAPK signaling pathway [124]. On human bone marrow mesenchymal stem cells, treatment with 10 μM of FA induced β-catenin expression due to HIF-1α-mediated miR-340-5p inhibition, improving cell osteogenesis [125]. On the other hand, Doss et al. demonstrated that treatment of RAW 264.7 monocytes and macrophages with 25, 50 and 100 μM FA decreased differentiation to mature osteoclasts mediated by the suppression of the RANKL-dependent NF-κB signaling pathway. Furthermore, it suppressed the bone resorption activity of mature osteoclasts by inhibiting the expression of genes such as TRAP, MMP-9 and cathepsin K, demonstrating its potential therapeutic use in bone loss disorders [126].

#### 5.2.9. Quercetin

Quercetin is a flavonoid present in fruits that performs many beneficial functions. In bone, quercetin has inhibitory effects on the formation, proliferation and maturation of osteoclasts and activating effects on osteoblastogenesis. The treatment of rat bone marrow mesenchymal stem cells with 5 μM quercetin elevated the mRNA levels of osteoblast-specific genes. Specifically, it stimulated Runx2, Osterix (OSX), OCN and OPN, thus promoting proliferation and osteogenic differentiation through miR-206/Cx43 [127]. In the same cells from ovariectomized rats, treatment with 1 μM of quercetin was able to promote cell proliferation, alkaline phosphatase (ALP) activity and the expression of osteogenic and angiogenic factors and reduce osteoclastogenesis by decreasing the expression of RANKL [128]. Furthermore, it has been proven that it protects against osteogenesis alterations induced by TNF-α by promoting cell proliferation and osteogenic differentiation [129]. In ovariectomized rats, treatment with microspheres loaded with 200 mM quercetin for 8 weeks induced osteogenesis and angiogenesis [128]. Another study in ovariectomized rats showed how the administration of 50 mg/kg once a day for 8 weeks increased bone mineral density and improved the biomechanical properties of bone [129]. Sun et al. demonstrated that treatment with 75 and 150 mg/kg/day of quercetin for 8 weeks in mice subjected to orchiectomy improved bone mineral quality and density through the modulation of bone metabolism through the GPCR6A/AMPK/mTOR pathway [130]. Due to the low bioavailability of quercetin, its possible topical administration is being studied. Pandit et al. found that films with transfersomes (a special type of very deformable liposome that can pass through pores much smaller than its size) loaded with quercetin (10 mg/kg) placed on the dorsal surface of rats with glucocorticoid-induced osteoporosis for 15 days decreased osteoclastogenesis and osteoblast apoptosis, an effect that induced an increase in the number of osteoblasts and bone mineralization [131]. Due to the importance of quercetin in regulating bone homeostasis, it could be used as a therapeutic agent to improve bone health.

#### 5.2.10. Kaempferol

Kaempferol, present in many fruits, vegetables and herbs, promotes osteogenesis and inhibits osteoclast differentiation. Kaempferol exerts a beneficial influence on the alterations in bone structure induced by estrogen deficiency. Nowak et al. demonstrated that, in ovariectomized rats, the administration of 5 mg/kg body weight of kaempferol for 8 weeks decreased bone turnover and increased Young’s modulus and bone perimeter. In rats with osteoporosis, induced by the same method, the administration of 5 mg/kg/day for 12 weeks of kaempferol increased bone density and improved osteoporosis by decreasing miR-10a-3p and increasing CXCL12 [132]. Furthermore, in female rats with glucocorticoid-induced bone loss and a perforation injury, the administration of 5 mg/kg/day kaempferol for 4 weeks reduced bone loss and improved its regeneration at the fracture site mediated by increased BMP2 [17]. In vitro, Sharma and Nam found that in primary and secondary cultures of human osteoblasts, treatment with 20 μM of kaempferol activated the Wnt pathway, an effect that induced osteogenic activity through the estrogen signaling pathway. These same authors described how the administration of 5 mg/kg weight of kaempferol for 6 days in mice with a small perforation in the tibia increased the expression of RunX2 and β-catenin, inducing osteogenesis [133]. Kim et al. treated RAW 264.7 with 50 μM kaempferol and observed that it inhibits osteoclast differentiation and bone resorption by blocking autophagy mainly by inhibiting p62/SQSTM1 expression and the activation of apoptosis [134]. On the other hand, treatment with 10 μM of kaempferol promoted osteogenesis in mesenchymal stem cells from the bone marrow of rats through the mediation of the SOX2/miR-124-3p/PI3K/Akt/mTOR axis, being able to alleviate the progression of osteoporosis [135]. These studies highlight the positive role of kaempferol in bone health and its possible role in pathologies associated with degenerative processes of the skeleton.

#### 5.2.11. Epicatechin

Catechins are the main polyphenols present in tea and are associated with an enhancement of osteoblastogenesis and suppression of osteoclastogenesis. Treatment for 2 weeks with 10 µM of Epigallocatechin-3-gallate (EGCG) in ovariectomized rats with tibial fractures accelerated bone matrix formation and increased BMP2 expression [136]. In mice with DEX-induced secondary osteoporosis, treatment with 5 mg/kg/day of EGCG improved bone mineral density and microstructure, improving bone quality [18]. In vitro, Han et al. demonstrated that pretreatment with 5, 10 or 20 μM of EGCG induces osteoblastic differentiation by decreasing the inhibitory effect of TNF-α on osteoblastic differentiation. Furthermore, EGCG increased the expression of lncRNA TUG1 which inhibited TNF-α-induced activation of the Hippo/YAP signaling pathway [137]. On human bone marrow stem cells, treatment with 1 and 10 μM increased BMP2, RunX2, ALP, osteonectin and osteocalcin mRNA levels, ALP activity and mineralization [137]. Chen et al. demonstrated that treatment of murine RAW 264.7 cells with 1 μM and 10 μM of EGCG decreased the RANKL/OPG ratio, decreasing osteoclastogenesis through the RANK/RANKL/OPG pathway, which suggests epicatechin as a possible therapeutic agent for the treatment of bone pathologies such as osteoporosis. These authors highlight the importance that this concentration can be easily achieved in daily tea consumption, postulating tea as a possible treatment for bone pathologies [138]. A prospective cohort study of 453,625 participants from the China Kadoorie Biobank (CKB) found that regular tea consumption is associated with a lower risk of fractures in both men and women [16]. However, the pure product was not used in the study, so the effect found could not be attributed only to epicatechin.

#### 5.2.12. Cyanidin

Cyanidin is one of the most common anthocyanins in fruits and vegetables such as red cabbage, blackberries, strawberries, blueberries and grapes. This molecule has a beneficial effect on bone health by stimulating the proliferation of osteoblasts and inhibiting osteoclastogenesis, protecting against bone loss. In ovariectomized mice, the consumption of 5 mg/kg of cyanidin chloride every 2 days for 6 weeks was associated with protection against bone loss [139]. The same authors demonstrated that in mouse bone marrow macrophages, treatment with 5 and 10 μM inhibited the activity of NF-κB, NFATc1 and MAPK, inhibiting the formation of osteoclasts [139]. In osteoblasts extracted from the hips of patients with osteoporosis, treatment with 200 and 100 μM of cyanidin-3-glucoside (C3G) promoted their proliferation and increased their mineralization [140]. On human osteoblast hFOB 1.19 with serum starvation, 1 μg/mL of cyanidin increased the proliferation and expression of SIRT1/3 and PGC1α and decreased the Bax/Bcl2, p53 and HDAC1 ratio. The col10α1:nlGFP/rankl:HSE:CFP transgenic medaka model overexpresses RANKL which induces an osteoporosis-like phenotype. In this model, 2 μg/mL of cyanidin for 5 days reduced RANKL-induced bone degradation [14]. On the other hand, various studies have been carried out with foods rich in cyanidins. Because the pure product was not used, the results cannot be attributed only to cyanidin.

In rats with diabetes-induced osteoporosis, administration of doses of 0.5, 1 and 2 g/kg/day of anthocyanin-rich black rice extract orally for 8 weeks improved bone loss caused by diabetes thanks to its inhibition of the bone turnover, suppression of bone marrow adipogenesis and upregulation of RunX 2 and the OPG/RANKL ratio [19]. Mahady et al. investigated the effects of a cyanidin-rich blackcurrant extract (CBE) on osteoblastogenesis on human hFOB 1.19 osteoblasts and in osterix/sp7:mCherry transgenic medaka. In hFOB, BCE increased osteoblast proliferation and reduced apoptosis by reducing the expression of Bax and p53 and altering the expression of HDAC1, HDAC3, SIRT 3 and PGC1α. In medaka osterix/Sp7:mCherry transgenic fish, treatment for 5 days with 10 mg/mL of BCE increased osteoblast proliferation by increasing osterix/Sp7 expression [141]. Supplementation with 240 mL of cherry juice, a fruit high in cyanidins, twice daily for 90 days reduced bone resorption in a cohort of postmenopausal women aged 65 to 80 years old [142]. Although more studies need to be carried out, cyanidins seem to have a positive effect on bone metabolism, postulating themselves as a good therapeutic agent in the treatment of osteoporosis.

#### 5.2.13. Carnosol

Carnosol (Car) is a phenolic diterpene found in plants of the rosemary, lavender and oregano families. Numerous studies have demonstrated that carnosol has inhibitory effects on osteoclastogenesis and protective effects on bone loss [143]. Treatment with 10 μM of Car for 5 days can inhibit RANKL-induced osteoclastogenesis by suppressing NFTAc1 in RAW 264.7 cells; this result also occurred in the MAPK signaling pathway (where Car inhibited the phosphorylation of p38 and ERK), NF-kB and Ca^2+^ in BMMcs cells [144]. In vitro [143], studies have shown that after 7 days, treatment with 0.25, 0.5 and 1 μM of carnosol reduced the formation of f-actin rings during osteoclastogenesis in a dose-dependent manner compared to the control group. The formation of f-actin rings is the precondition for the formation of osteoclasts. The results of this study also demonstrated how Car decreased proteins related to osteoclastogenesis, such as TRAP, cathepsin K and MMP-9. To evaluate the activity of osteoclasts and osteoblasts, serum levels of Tracp5b, cTX-1 and IL-6 were measured for 6 weeks, which, after treatment with Car (10 mg/kg), decreased significantly [143]. On the other hand, the protection of Car on bone loss in murines was demonstrated, where they suggested that the consumption of 10 mg/kg of carnosol for 10 days showed protection against bone loss induced by lipopolysaccharides, also improving BV/TV, Tb. N and Tb.Th [145].

#### 5.2.14. Apigenin

Apigenin (Api), is a plant flavone found abundantly in fruits and vegetables, such as onions, oranges or wheat sprouts; aromatic plants (chamomile, mint, parsley) and honey. Api plays an important role in human mesenchymal cells (hMSCs) such as osteoblastic cells (MC3T3-E1) since it facilitates osteogenic differentiation [146]. In addition, it participates in the formation and development of the skeleton. Regarding human MSC cells [146], researchers have investigated the effect of Api on early osteogenesis, with a dose of 0.5 μM, which managed to modulate the activity of ALP, BMP-2, OPN and OCN; therefore, Api would have a modulating effect on osteogenic differentiation. According to this dose, the expression of Wnt/β-catenin receptors (FZ, LRP5, Ror, and PTK7) was promoted by Api, thus inactivating the Wnt/β-catenin pathway in MSCs. In this same study, a femoral fracture model with mice was used to evaluate the role of apigenin on bone formation [146], from which they suggested that a dose of 45ng/kg of apigenin increased the number of cells and mineralization at the fracture site in weeks 4 and 6 of treatment. The addition of apigenin improves mineralization in hFOB 1.19 osteoblastic cells and Saos-2 osteosarcoma cells, being more evident in Saos-2 cells since it activates the TNAP protein from 1 μM [147].

#### 5.2.15. Omega-3

Omega-3 acid is a polyunsaturated fatty acid that can be found in fish oil and vegetable oils. It has health benefits against various diseases, including cardiovascular, neurodegenerative, cancer and bone diseases [148]. Some studies propose that omega-3 acid improves bone quality by preventing bone breakdown and increasing mineralization. Researchers [148] propose that omega-3 supplementation helps bone preservation in older women at risk of osteoporosis and, on the other hand, interferes with the pathological calcification of vascular cells and cancer cells. According to the data shown, omega-3 supplementation should be included with chemotherapy for cancer patients, as it can prevent the osteoblastic potential of breast cancer patients. The effects of omega-3 fatty acid on bone turnover markers in postmenopausal women were studied in a meta-analysis [149] in which it is shown that its use/application significantly reduces serum OCN in a cohort of studied patients.

#### 5.2.16. Gingerol

Gingerol, a polyphenol present in ginger, presents different effects according to what is described by different studies. It has been shown that 10-gingerol at 2.5 µM suppresses osteoclastogenesis in RAW 264.7 cells in vitro. This effect is due to the suppression of the osteoclast differentiation activator RANKL in macrophages. The same authors study the regeneration of scales in goatfish, which present great functional similarity to human bones so that once removed, osteogenesis is activated, including the differentiation of osteoblasts, the deposition of the matrix and its mineralization. It has been described how 10-gingerol (0.1 μg/mL) also suppresses osteoclastogenesis [150]. On the other hand, another structure mediated by these molecules, 6-gingerol, was studied on human osteoblastic-type cells (MG63 cells), in which inflammation was induced by TNF-α, showing that the administration of 50 µM of this polyphenol stimulated the proliferation and differentiation of osteoblasts, also increasing ALP activity, the formation and improvement of enhanced mineralized nodules and the reduction of TNF-alpha-induced inflammation [151]. This effect was also verified with an ex vivo model using mouse bone marrow cells, where it was demonstrated that with the administration of 1.25 to 10 µM of 6-gingerol, the differentiation of osteoclasts was inhibited through the suppression of RANKL expression in osteoblasts and with the reduction of PGE_2_ levels, which suggests its potential use as a treatment for inflammation produced by excessive amounts of PGE_2_.

#### 5.2.17. Gallic Acid

Present in tea leaves and other plants, gallic acid (GA) is an organic polyphenol with different biological functions. Regarding the studies carried out with gallic acid, its osteogenic capacity has been described in vitro. Two works showed that the addition of this polyphenol to cell support structures such as chitosan (Q) (111 mg GA/g Q) [151] or scaffold models (28 mM GA) [152] stimulated the differentiation of mesenchymal stem cells into osteoblasts. In the first study, this was achieved through the Wnt/β-catenin signaling pathway, and in the second study, in addition to increasing the proliferation of osteoblasts, a decrease in their apoptosis was observed. Also, in pathologies such as periodontitis, the effectiveness in the differentiation of stem cells into osteoblasts has been proven, from the use of gallic acid (10 mM) [152].

#### 5.2.18. β-Carotenes

β-Carotenes (βC) are a natural pigment found in vegetables and fruits, it is the main source of vitamin A and has been used for the treatment of cancer and cardiovascular diseases, as well as for improving immunity. Recently, these natural pigments have been proposed to see the effect they have on bone resorption, bone mineral density and osteoclast formation. Numerous studies suggest the role of βC in improving osteoporosis. βC intake was associated with an improvement in overall bone mineral density thereby reducing the risk of fracture [153]. This same effect was observed [154] in a meta-analysis after treatment with 1.76–14.30 mg/day of βC in men and women, where it was suggested that this intervention reduced the risk of any fracture by more than 20% and the risk of fracture by 95%. In mice, a diet with 0.025% of βC also reduced alterations in bone mineral density after 3 weeks of treatment [155]. There is also evidence of the use of βC as osteogenic material incorporated into bone tissue engineering. This was achieved through the ability to express RunX2, SOX9 and osteonectin, generating an osteoinductive effect on the differentiation of MSC into osteoblasts [156].

#### 5.2.19. Luteolin

Luteolin (LUT) is a flavone compound that is present in substantial amounts in many widely consumed foods, such as broccoli, onions, carrots, peppers, cabbage and apples. LUT promotes a protective effect on osteoporosis, inhibiting it through various pathways [157].

A study on glucocorticoid-induced osteoporosis in osteoblastic cells from mice (MC3T3-E1) evaluated the effects of LUT (0.05, 0.1 and 0.2 μM) and DXM (20 μM) for 48 h. The results showed that LUT improved osteoblast osteogenic markers RunX2, OSX, col-1 and OCN. This same study suggested that LUT (0.05, 0.1 and 0.2 μM) decreased the levels of ROS generation in MC3T3-E. On the other hand, another study states that LUT at concentrations between 1 and 10 μM attenuates oxidative stress biomarkers (nitric oxide, TNF-α and IL-6) [158].

Within its anti-inflammatory and preventive properties [159], the effect of LUT on experimental periodontitis was studied by treating 28 rats with a dose of 50 mg/kg and 100 mg/kg. The effects at both doses showed increases in the expression of TIMP-1, BMP-2 and OPG and decreases in the expression of MMP-8, RANKL and inducible nitric oxide. With these data, they concluded that LUT prevents periodontal disease by reducing bone loss and inflammation [159].

To verify the effect of LUT on osteoporosis, a rat model was treated with 10 mg/kg and another group with 50 mg/kg, in these two models an increase in BMD, BV/TV and Tb.N was seen after 8 weeks. In this same study, the osteogenic differentiation of mouse BMNSCs was also examined, through the protein levels of the BMNSCs and the PI3K/Akt pathway. Another study [160] regarding the effects of LUT in osteoporosis showed an increase in the expression levels of collagen I, osteopontin and RunX2 proteins in BMSCs, with doses of 0.5, 1 and 5 μM and a regulation of the activation of the PI3K/Akt pathway.

Table 2 summarizes the effects of the bioactive compounds, including the studied doses and main sources mentioned in this article

### 5.3. Physical Therapy

Regarding the impact of physical therapy on bone tissue damaged by osteoporosis, there is currently abundant evidence and expert guides with clinical orientations [161], which are based on effective results in improving parameters such as the BMD of the lumbar spine and femoral neck [162]; however, the specific mechanism by which these effects are exerted has not yet been described, although one of the hypotheses is based on “mechanotransduction”, which is discussed in this article. On the other hand, a recent systematic review [163] analyzed three clinical investigations in relation to the effect of physical activity as a therapeutic strategy in improving bone biomarkers and BMD of the subjects under study. Regarding biomarkers, all three studies included serum alkaline phosphatase, a marker of osteoblastic activity and therefore bone formation [164,165,166] Roghani et al. and El-Mekawy et al. included serum calcium, and Arazi et al. looked at 25-hydroxyvitamin D (25OHD), which is essential in the bone mineralization process, with all of them significantly improving with their physical activity interventions. The best effect was shown by Roghani et al., with submaximal aerobic exercise with a weighted vest in postmenopausal women. On the other hand, El-Mekawy et al. reported an increase in BMD in the femoral neck and lumbar spine with the highest score for the weight-bearing exercise group.

In relation to the different physical modalities and their potential effects on osteoporosis, there are emerging preclinical studies such as one that describes how electroacupuncture improved bone microstructure in senile osteoporotic rats and describes autophagy as a possible mechanism [167]. Similarly, another preclinical study with osteoporotic ovariectomized female Wistar rats showed that treatment with radial extracorporeal shock waves and ultrasound improved trabecular bone microarchitecture and bone strength, but not electrical stimulation. Shockwaves decreased osteoclast activity and RANKL expression. Ultrasound exposure increased the activity of osteoblasts and β-catenin-positive cells and decreased sclerostin-positive osteocytes [168]. Regarding the pulsed electromagnetic field, a recent meta-analysis showed that it could be used as a complementary strategy to the treatment in postmenopausal women with osteoporosis, by significantly improving lumbar and femoral BMD, also improving biochemical markers such as alkaline phosphatase, osteocalcin, which is a protein produced by osteoblasts and therefore an indicator of greater bone formation and a collaborator in bone mineralization process, and bone-specific alkaline phosphatase, specific marker of bone formation [169]. Despite being an emerging field of research and the specific mechanisms being unknown, the different non-invasive physical therapeutic modalities are potentially a therapeutic strategy; however, much remains to be understood.

## 6. Conclusions

In addition to pharmacological interventions, lifestyle changes might be useful for the prevention and treatment of osteoporosis. These include reducing alcohol consumption, taking nutritional supplements such as vitamin D and not smoking [88]. Tobacco reduces bone density, causing a loss in bone quality and increasing the number of fracture cases [170]. Regarding alcohol, it has been shown that continued consumption of large amounts of alcohol negatively affects bone mass since there is an overactivation of osteoclasts with the consequent loss of mineralized matrix [171]. As previously mentioned, the mechanical load that allows for better bone regulation, and that can be achieved through movement or external load, positions exercise as a powerful preventive and management tool during the treatment of osteoporosis [172]. Another lifestyle factor to take into account is physiological stress (especially chronic), and its deregulation signal in glucocorticoids has a profound impact on inflammation and acts directly on bone cells and on the transcription of genes such as NF-κB. Therefore, physiological stress could negatively impact bone health through endogenous glucocorticoid modulation. Likewise, catecholamines could act on bone health through β-adrenergic receptors in osteoblasts or osteoclasts and the creation of reactive oxygen species (ROS) [173].

Regarding the beneficial dose of vitamin D, studies have not established a range of serum 25(OH)D concentrations but the international consensus is that the optimal requirements for vitamin D (25(OH)D) are those that allow reaching plasma levels greater than 30 ng/L (75 nmol/L), considering that baseline 25(OH)D, body mass index, ethnicity, type of vitamin D (D2 or D3) and genetics affect the response of serum 25(OH)D to vitamin D supplementation, but institutions and scientific societies have published their recommendations for vitamin D intake that range from 400 to 1000 IU/day of vitamin D3 (10 to 25 μg/day) for an average adult. More studies are needed on babies, children and pregnant and lactating women [174,175,176]. Vitamin D is metabolized as 25(OH)D in the liver, which is metabolized again into 1,25(OH)2D in the kidney, being the most biologically active form of vitamin D. In the blood, the most abundant is 25(OH)D, which is used as a marker of nutritional status due to its long half-life (2–3 weeks). Older adults have greater requirements for vitamin D since aging decreases the synthesis of vitamin D and increases the resistance of the action of 1,25(OH)2D in the intestine; this added to the difficulty of making dietary changes in these groups. Additionally, various meta-analyses of randomized controlled trials on the effects of vitamin D on fracture risk, falls or osteoarthritis, indicate that 1000 IU daily should be recommended in patients at increased risk of vitamin D deficiency [177].

## Figures and Tables

**Table 1:** Prevalence of osteoporosis in male and female population by continent [4].

Continent	Prevalence (%)	
	Men	Women
Asia	11.7	24.3
Europe	9.7	19.8
America	8.5	15.1
Africa	No Data	42.4

**Table 2:** Summary of the effects of various bioactive compounds present in diet on osteoporosis. ↑ indicates increase or upregulation and ↓ indicates decrease or downregulation.

Compound	Source	Dose	Relevant Effect
Caffeic Acid	Coffee, tea, wine, blueberries, apples and honey.	5–10 µM in cells. 0.1–5 µM in mice.	↑ Osteoblast proliferation. ↑ Bone mineralization. ↓ Osteoclast proliferation.
Resveratrol	Grapes, wine, blueberries, blackberries and peanuts.	75 mg in humans. 625 µg/kg and 40–50 mg/day, rats. 10 µM in cells.	↑ Bone mineral density and ↓ risk of fracture. ↓ Osteoclastogenesis and beneficial effect through miR-193a/SIRT-7 mediated NF-κB alleviating osteoporosis. Improved oxidative stress and ↓ expression. mRNA osteoclast-specific enzyme ↓ miR-193a.
Tangeretin	Citrus fruits.	Cells and animals. 5 µM in cells. 30 µM in cells.	Silencing miR-137-3p, improves osteogenesis enhancing RunX2. Reverses miR-137, knockdown on osteogenic promotion. Suppresses bone resorption factors associated with inflammation.
Urolithin	Intestinal microbial metabolite of ellagic acid and ellagitannins present in pomegranate, berries and walnuts.	10–50 mg/kg/day in ovariectomized mice. 5–10 µM in cells.	↓ Systemic bone loss. Inhibits osteoclast differentiation and suppresses expression of osteoclast-related genes. Modulate macrophage polarization. ↓ Inflammation. ↓ RANKL-triggered osteoclastogenesis ↓ Osteoclast-related genes.
Oleocanthal	Compound isolated from olive oil.	25–50 µM in cells. Animals.	Anti-inflammatory. Attenuates loss of bone mass.
Naringenin	Citrus fruit, grapefruit.	10–15 µM in cells. 20 mg/kg/day in female ovariectomized rats.	Improves synthesis of osteoprotegerin. Increases osteocalcin. Stimulates stem cell differentiation into osteoblasts. RANKL inhibition. ↑ Bone density.
Curcumin	Curcuma longa species.	110 mg/kg, ovariectomized rats 80 mg in humans (postmenopausal women).	↓ Bone loss. Inhibits osteoclastogenesis. Activates biomarkers of bone turnover.
Ferulic Acid	Cereals, vegetables, fruits and nuts.	20–30 mg/kg on rats. 25–100 µM in cells.	Inhibits differentiation and formation of osteoclasts and stimulates osteoblast differentiation. ↑ Bone mineral density. ↓ Bone loss. ↓ RANKL. ↓ Mature osteoclast differentiation and suppressed bone resorption.
Quercetin	Fruits.	1–5 µM in cells. 200 mM on ovariectomized rats. 50 µM on ovariectomized rats.	Inhibits formation, proliferation and maturation of osteoclasts and activates osteoblasts. ↑ Osteoblasts genes. ↑ Osteogenesis. ↑ Bone mineral density.
Kaempferol	Fruits, vegetables and herbs.	5 mg/kg on ovariectomized rats.	↑ Osteogenesis and inhibits osteoclast differentiation. ↑ Bone density. ↓ Bone loss.
Epicatechin	Tea.	10 µM on ovariectomized rats. 5 µM on mice. 5–20 µM in cells	↑ Osteoblastogenesis and ↓ Osteoclastogenesis. Accelerates bone matrix formation. ↑ Bone mineral density and microstructure (↑ bone quality). Induces osteoblastic differentiation. ↓ Osteoclastogenesis.
Cyanidin	Fruit and vegetables.	5 mg/kg in ovariectomized mice. 5–10 µM in cells. 2 µg/mL in humans.	↑ Osteoblasts proliferation and ↓osteoclastogenesis. Protection against bone loss. Inhibits osteoclast formation. ↓ RANKL.
Carnosol	Rosemary, lavender and oregano plants.	0.25–1 µM in cells. 10 mg/kg in mice.	Inhibits osteoclastogenesis and protects against bone loss. Protection against bone loss.
Apigenin	Fruits and vegetables (onions, oranges and aromatic plants) and honey.	0.5 µM in cells. 45 ng/kg in femoral fracture mice model.	Effect on osteogenic differentiation. ↑ Number of cells and mineralization at the fracture site.
Omega-3	Fish and vegetable oils.	Postmenopausal, meta-analysis.	Improves bone quality by preventing bone breakdown and increasing mineralization. Reduces osteocalcin serum.
Gingerol	Ginger.	2.5 µM in cells. 50 µM in cells.	Suppresses osteoclastogenesis. Stimulates proliferation and osteoblast differentiation.
Gallic Acid	Tea leaves and other plants.	Cells.	↑ Stem cell differentiation in osteoblasts. ↓ Osteoblast apoptosis.
β-Carotenes	Vegetables and fruits.	1.76–14.30 mg/day, meta-analysis (men and women). 0.025% in mouse diet.	Improves overall bone mineral density ↓ Risk of fracture. ↓ Alterations in bone mineral density.
Luteolin	Broccoli, onions, carrots, peppers, cabbage and apples.	0.05–2 µM in cells. 10–50 mg/day on rats.	Improves osteoblast osteogenic markers and ↓ ROS. ↑ Bone mineral density.

## Data Availability

Data sharing is not applicable.

## Appendix A

As described in the introduction, osteoporosis is a chronic disease characterized by a loss of bone density, resulting in an increased risk of bone fractures, thus making osteoporosis a major cause of disability and even premature death in the elderly.

For this article, we chose to focus on the risk factors of the disease and how certain compounds present in diet as well as an active lifestyle could reduce these risks. A comprehensive literature search was conducted to identify studies showing the relationship between diet and osteoporosis. The search was performed in databases such as PubMed, Web of Science and Scopus in the last 5 years. The keywords used included the different polyphenols or compounds mentioned in the article in combination with “osteoporosis” in the title or abstract. We also searched for articles using the keywords “risk factors”, “prevention”, “lifestyle” or “physical exercise” in combination with “osteoporosis” in the title or abstract.

Following the outlined searches, articles were chosen based on their relevance to the objective of this review. The abstracts of all the articles with relevant titles were read. Through analysis of the abstract, the articles showing relevance to our study were read in their entirety.

The articles providing clear information on the risk factors and prevention strategies related to diet and osteoporosis and their action mechanisms were included in this review. Only studies reporting original data from human studies, such as observational studies, randomized controlled trials, animal studies or in vitro studies published in the last 5 years were included (2019–2024). All review articles, meta-analyses and non-peer-reviewed articles were excluded from this review.
